# Risk Factors Associated With Hyperammonemia Following Unprovoked Convulsive Seizures

**DOI:** 10.7759/cureus.8504

**Published:** 2020-06-08

**Authors:** Nurose Karim, Giana Dawod, Nicholas D Henkel, Ajaz A Sheikh

**Affiliations:** 1 Neurology, University of Toledo Medical Center, Toledo, USA; 2 Neurology, University of Toledo College of Medicine, Toledo, USA

**Keywords:** convulsive seizures, hyperammonemia, metabolism, neurophysiology

## Abstract

This report describes a unique case of recurrent transient hyperammonemia (THA) following a first-time occurrence of generalized tonic-clonic seizure in a young adult, who went on to develop post-stroke epilepsy. Although this phenomenon has been described in recent literature, we report not only the highest initial ammonia level to date, 549 µmol/L, but we also document serial trends of the ammonia levels at multiple admissions for the same patient for the management of breakthrough seizures. Interestingly enough, persistence of the elevation of the ammonia levels was accompanied by no other significant metabolic derangements, unlike reported in similar cases. Of prior studies, high ammonia levels have been reported in the context of alcohol-induced seizures, with resolution of ammonia levels within eight hours. Here, we highlight the importance of medication compliance, as well as the need for serial ammonia levels for improving patient outcomes, with the knowledge that ammonia accumulation leads to potential irreversible neurotoxicity. Additionally, we completed a systematic literature review on data pertaining to the risk factors associated with hyperammonemia following unprovoked convulsive seizures in an effort to analyze our case in the context of the existing literature. Our objective is to ultimately understand the utility of serial ammonia levels for unprovoked convulsive seizures in the context of the patient's initial presentation, and whether treatment of these episodes of hyperammonemia can significantly alter outcomes.

## Introduction

Although the diagnosis of a seizure relies heavily on the clinical history, the initial workup for patients presenting to the emergency department (ED) includes both imaging and serum markers. Our collective interpretation of these results and their trends assists us in better management of the patient’s neurological disturbance. Many physical, electrical, and biochemical abnormalities tend to surface after seizure activity, with serum ammonia levels included as part of the initial workup. Ammonia, a precursor of glutamine, is normally metabolized in the liver to urea, which is easily excreted. In the context of prolonged seizure activity, vigorous skeletal muscle contraction leads to increased levels of ammonia as adenosine triphosphate (ATP) is depleted, overwhelming the liver’s ability to aid in extraction of the ammonia from the bloodstream [1]. A buildup of ammonia, or an inability to clear it appropriately, as documented in acute liver failure, cirrhosis, renal failure, diabetic ketoacidosis, or acute alcohol intoxication, can lead to altered mental status and cognitive deficits [2]. Ammonia remains neurotoxic, with high levels leading to its crossing of the blood-brain barrier, where it is converted to glutamine, an osmotic agent that promotes cerebral edema [1,2]. Recently, studies have demonstrated an association between transient hyperammonemia (THA) after a seizure and postictal confusion. Here, we present a case of a patient who presented with the highest serum ammonia levels to date following an unprovoked convulsive seizure. In an effort to understand our patient's case within the context of the literature present with regards to hyperammonemia related to prolonged seizure activity, we conclude the case presentation with a discussion on our findings from our systematic literature review.

## Case presentation

A 27-year-old male presented to the outside hospital ED complaining of several days of speech difficulty and cognitive dullness. While in the ED, he had a witnessed first-time seizure. Brain MRI confirmed an acute left middle cerebral artery stroke, and further workup showed a patent foramen ovale. His seizures were described as the patient beginning to stare off, followed by right head version, progressing to generalized clonic semiology. Although the electroencephalograms (EEGs) have shown left fronto-temporal interictal epileptiform discharges, video-EEG recording showed electrographic seizures arising from right temporal region, indicating bihemispheric epileptogenicity. After his first seizure, his ammonia level was initially 549 μmol/L, with slightly elevated liver enzymes, aspartate aminotransferase (AST) and alanine aminotransferase (ALT) being below 100 units/L, and normal renal function. At that time, he was started on phenytoin 200 daily and valproic acid 250 twice a day. Thereafter, he was readmitted multiple times for breakthrough seizures due to medication non-compliance. On most of these presentations, the ammonia levels were high with an otherwise normal metabolic workup. Table 1 shows details regarding the ammonia levels, as well as the other metabolic markers, upon the patient’s numerous admissions.

**Table 1:** Serial lab values of the patient during several visits “-” in the table implies that the test was not done. BUN, blood urea nitrogen.

Visits	Initial ammonia level (μmol/L)	AST (U/L)	ALT (U/L)	Pyruvate	Lactate (mmol/L)	Orotic acid (mmol/mol)	BUN (mg/dL)	Creatinine (mg/dL)
12/30/2015	549	43	64	-	1.7		14	0.97
12/31/2015	443	-	-	-	-		10	1.28
12/31/2015 (evening)	12	48	24	0.15 (0.08-0.16) mmol/L	1.6		9	1.43
1/1/16	22	-	-	1.3(0.7-1.4) mg/dL	-		6	1.07
12/31/2018	529	-	-	-	-		15	1.12
1/1/2019	60	-	-	-	-		-	-
1/6/2019	71	-	-	-	-		-	-
8/21/2019	-	70	25	-	-		17	1.42
9/5/2019	35	-	-	-	-		13	0.92
1/3/2020	-	-	-	-	-	0.8	-	-

A review of his medications showed that he had been on phenytoin, valproate, lacosamide, levetiracetam, and oxcarbazepine. Valproate has been associated with hyperammonemia in the postictal state; however, this does not explain our findings since he was on it for less than a month. His ammonia levels remained elevated past eight hours, which is the longest reported period of postictal ammonia levels. This raises an important question of whether the hyperammonemia was the cause of seizure, or its effect. Hyperammonemia can result from excessive production or impaired elimination of ammonia, seen in both liver and renal failure; yet the patient did not have labs representative of either. Upon screening for urea-cycle disorders, results were normal.

## Discussion

Ammonia is a toxic metabolite in the body. It is produced by the breakdown of amino acids and other compounds that contain nitrogen. It can exist as both ammonia (NH_3_) and, in the ionic form, as ammonium ion (NH_4_^+^). Majority of ammonia under physiological conditions exists as NH_4_^+^, and only about 1.7% of total ammonia presents as NH_3_ at a pH of 7.4. The primary source of ammonia is glutamine, which gets excreted in the urine. Hyperammonemia, defined as ammonia concentrations in the systemic circulation above or equal to 65 μmol/L, can occur in conditions with excessive breakdown of muscles, liver failure, or failure of excretion [1].

Some of the non-hepatic causes of THA are reversible, if recognized and treated early. One of the rare but treatable causes is urinary tract infection with urea-hydrolyzing organisms, which hydrolyze urinary urea to ammonium. It is converted to ammonia in alkaline pH [2]. 5-Fluorouracil (5-FU), which is used in the treatment of various malignancies, including gastrointestinal tract, breast, head and neck, and ovaries, is another well-known cause of THA [3]. Koenig et al. proposed that fluoroacetate, one of the metabolites of 5-FU, suppresses the Krebs cycle and causes dysfunction in the ATP-dependent urea cycle, leading to transient accumulation of ammonium [4].

The relationship between hyperammonemia and myopathy is complex. It includes the activation of autophagy leading to an increase in muscle protein breakdown, alterations in energy supply required for ATP‐consuming protein synthesis, and the upregulation of myostatin, a negative regulator of muscle mass, leading to a suppression of muscle protein synthesis [5-7].

THA following generalized convulsions was first reported in a case series by Yanagawa et al. in 2008 with vigorous muscle activity as the proposed mechanism [8]. Two larger cohort studies, one retrospective and one prospective study, found an association between THA and generalized convulsive seizures, and its utility in differentiating epileptic seizures from non-epileptic events [9-11]. With a cutoff value set at 46.4 μmol/L, possessing a sensitivity of 53% and specificity of 90%, ammonia levels can be confidently used as a confirmatory test of “high clinical significance” in diagnosing generalized convulsions [11].

Therefore, venous ammonia level has been frequently used in the ED when there is a concern for seizure activity, as it is a cheap, non-invasive, and readily available test. It can be used as a blood marker to differentiate epileptic seizures from non-epileptic events. We should also keep in mind that hyperammonemia itself can induce seizures secondary to brain edema, and the presence of increased extracellular glutamate is caused by a combination of both increased release and decreased uptake by perineuronal astrocytes. Other serum markers used to differentiate convulsive seizures from non-convulsive/psychogenic seizures include prolactin and creatinine kinase (CK). However, the utility of these markers remains limited, given that prolactin is only reliable if checked within 20 minutes of seizure onset (sensitivity 60.0%, specificity 96%), due to its rapid degradation [12]. Peak serum CK levels after a seizure also vary from upon ED arrival to two days after the seizure. Therefore, ammonia can be considered a reliable maker as it lacks the limitations of other blood markers [13].

So far, previous studies have concluded THA to be resolved in less than eight hours [14]. In our case, the levels were much higher than those previously reported, recurrent with each episode of breakthrough seizure, lasted more than 48 hours, and were with a relatively normal metabolic panel.

In an effort to understand our patient’s case in the context of the literature, a systematic literature review was completed in conjunction with our case report. Of interest, several studies mentioned various presenting ammonia levels for patients with seizures, in addition to other variables, such as initial Glasgow Outcome Score, specific diagnosis, and use of certain medications. Here, we have completed the first systematic literature review in an effort to find an association between hyperammonemia at presentation for an unprovoked convulsive seizure and gender, with the hope of elucidating the true diagnostic value of a serum ammonia level.

A systematic review of the PubMed database was completed using the Preferred Reporting Items for Systematic Reviews and Meta-Analyses (PRISMA) guidelines [15]. Keywords used to define the search included “Seizure” and “Hyperammonemia”. A total of 302 studies were found. Case reports, studies that had patients on valproic acid, literature reviews, pediatric, non-English, and animal studies were excluded. Studies that focused on patients with underlying metabolic disorders or malignancies that predisposed them to seizures were also excluded.

Four studies met inclusion criteria [5,14,16,17]. From our literature review, the hyperammonemia cohort had a greater proportion of males than females (77.4% v. 22.5%, respectively, p < 0.0001). However, there was no difference in the proportion of males or females in the non-hyperammonemia cohort (51.8% v. 48.1%, respectively, p = 0.38978). Two studies were used to study the difference in average ammonia levels in patients with unprovoked seizures. As expected, the hyperammonemia patients with convulsive seizures had a significantly greater ammonia level (p = 0.02561) than the non-hyperammonemia cohort. Due to limitations in the number of studies, an analysis could not be conducted on differences in ammonia levels between convulsive and non-convulsive seizures.

In light of these findings, our case aligns with the risk factors reported to be significant in our literature search, with both elevated ammonia levels and the male gender being the greatest risk factors for THA following unprovoked convulsive seizures.

## Conclusions

Our literature search concludes that male patients presenting with unprovoked convulsive seizures have higher ammonia levels. While this review confirms the diagnostic role potential ammonia levels have, it is limited by the lack of studies that have followed these patient cohorts over time. Further analysis is warranted to see how the elevation of ammonia levels affects patient functionality outcomes and readmission rates.
